# Identification of RE1-Silencing Transcription Factor as a Promoter of Metastasis in Pancreatic Cancer

**DOI:** 10.3389/fonc.2019.00291

**Published:** 2019-04-16

**Authors:** Haoyi Jin, Peng Liu, Lingming Kong, Xiang Fei, Yang Gao, Tianyu Wu, Defeng Sun, Xiaodong Tan

**Affiliations:** ^1^Department of Surgery, Shengjing Hospital of China Medical University, Shenyang, China; ^2^Thyroid and Pancreatic Surgery Ward, Shengjing Hospital of China Medical University, Shenyang, China

**Keywords:** RE1-silencing transcription factor, metastasis, pancreatic cancer, weighted gene co-expression network analysis, prognosis

## Abstract

Pancreatic cancer is characterized by its rapid progression and early metastasis. This requires further elucidation of the key promoters for its progression and metastasis. In this study, we identified REST as the hub gene of a gene module which is closely associated with cancer stage by weighted gene correlation network analysis. Validation with the TCGA database, western blot analysis of human pancreatic cancer cell lines (AsPC-1, Capan-2, SW-1990, and PANC-1) and immunohistochemical analysis of paraffin-embedded pancreatic cancer tissue sections showed that REST was enriched in tissue samples of advanced stage and metastatic phenotype cell lines. Survival analysis with the TCGA database and our own follow-up data suggested that patients with higher expression level of REST showed worse overall survival rate. *In vitro* functional experiments suggested that knockdown of REST suppressed proliferation, migration, invasion and epithelial-mesenchymal transition of AsPC-1 and PANC-1 cells. *In vivo* experiments (a subcutaneous BALB/c nude mouse model and a superior mesenteric vein injection BALB/c nude mouse model) suggested that knockdown of REST suppressed growth and metastasis of xenograft tumor. Finally, we investigated the underlying molecular mechanism of REST and identified REST as a potential downstream target of MAPK signaling pathway. In conclusion, our results of bioinformatic analysis, *in vitro* and *in vivo* functional analysis suggested that REST may serve as a promoter of metastasis in pancreatic cancer.

## Introduction

Pancreatic cancer, ranked fourth in cancer-related mortality, is characterized by its rapid progression and early metastasis (1). Patients are hardly cured by surgical resection due to the existing local invasion and distant metastasis (2). Elevating the overall survival rate for pancreatic cancer requires further elucidation of the underlying molecular mechanism of its metastasis. Epithelial-mesenchymal transition (EMT), especially type III EMT, is reported to be one of the primal steps for cancer cells' local invasion and distant metastasis (3, 4). It is a complicated process in which cancer cells transit from a rather dominant epithelial phenotype to a more invasive and metastatic mesenchymal phenotype (5). This process is characterized by alterations in the expression level of multiple proteins leading to the loss of cell junctions and apical-basal polarity in cancer cells. Therefore, identification of key genes regulating EMT and cancer metastasis possesses great clinical significance (6). These genes could be predictive biomarkers for cancer metastasis and novel therapy targets for anti-cancer drugs.

The Cancer Genome Atlas (TCGA) is a comprehensive cancer genomic database (7). All of its cancer tissue and matched normal tissue samples were collected and further analyzed under the same standard procedure. Furthermore, it contains detailed clinical data of these samples including information like cancer stage and follow-up data. Weighted gene correlation network analysis (WGCNA) is an R language package capable of identifying clinically interesting gene modules from genomic data and further correlates them with corresponding clinical phenotype (8). Therefore, WGCNA analysis of data from the TCGA database holds promising potential of identifying key genes for cancer metastasis (9).

RE1-silencing transcription factor (REST) functions as the transcriptional repressor of neuronal genes in non-neuronal cells to restrict the expression of neuronal genes in nervous system (10–12). One thousand eight hundred ninety-two genes in human genomes and 1,894 genes in mouse genomes have been reported as direct targets of REST by a Genome-wide analysis of REST's target genes (13). Multiples studies have reported the aberrant expression or mutation of REST in different cancers. Interestingly, REST showed diverse role in the progression of these cancers. For instance, REST may function as tumor suppressor in prostate cancer, small cell lung cancer, colon cancer, Wilms tumor, and breast cancer while function as oncogene in medulloblastoma and glioma (14–21). This rather contradictory results may partly explained by the fact that REST has multiple splice variants in different cancers. For example, in small cell lung cancer cells, the expression of REST is undetectable while a small cell lung cancer specific isoform of REST is highly expressed (18). However, studies on the role of REST in pancreatic cancer progression has not been reported.

In this study, we identified a gene module of 95 genes which is closely correlated with pancreatic cancer stage through analyzing genomic and clinical data in the TCGA database by WGCNA analysis. Protein-protein interaction (PPI) network analysis of the gene module identified REST as the hub gene. Validation with the TCGA database, western blot analysis of human pancreatic cancer cell lines, and immunohistochemical (IHC) analysis of paraffin-embedded pancreatic cancer tissue sections showed that the expression level of REST was higher in advanced stage pancreatic cancer tissue samples and metastatic phenotype cell lines. Survival analysis with clinical data from the TCGA database and our own follow-up data suggested that patients with higher expression level of REST showed worse overall survival rate. Results of *in vitro* and *in vivo* experiments indicated that REST knockdown suppressed metastatic ability of the PANC-1, AsPC-1 cell lines, and nude mice tumor model. Finally, results of western blot analysis suggested that REST may serve as a downstream target of mitogen-activated protein kinase (MAPK) signaling pathway.

## Materials and Methods

### Ethics Approval and Consent to Participate

The current study was approved by local ethics committee of the affiliated Shengjing Hospital of China Medical University (animal studies-2017PS006K, tissue sample collection-2017PS24K). All animal handling procedures were performed in accordance with standard procedures of the local ethics committee. Thirty-eight qualified paraffin-embedded pancreatic cancer tissue sections with follow-up data were collected from the local pathological department of Shengjing Hospital between 2014 and 2016.

### Bioinformatic Analysis

Expression and clinical data of pancreatic cancer in the TCGA database were downloaded and further analyzed with R language package WGCNA. Identification of the key gene module for cancer stage with WGCNA analysis was performed in accordance with the official tutorials. PPI network of the identified gene module was visualized with Cytoscape software (v3.6.1) and its internal app cytoHubba. Gene Ontology (GO) analysis and Kyoto Encyclopedia of Genes and Genomes (KEGG) pathway analysis of the identified gene module was performed with the open-access database Database for Annotation, Visualization and Integrated Discovery (DAVID, https://david.ncifcrf.gov/) (22–24). The expression level of REST in different cancer stages was analyzed the with online analysis platform UALCAN (http://ualcan.path.uab.edu/index.html) (25). Survival analysis of patients with different expression level of REST was analyzed with Kaplan-Meier Plotter (http://kmplot.com/analysis/index.php?p=service&cancer=pancancer_rnaseq)(26).

### Cell Lines and Cell Culture

Human pancreatic cancer cell lines AsPC-1, Capan-2, SW-1990, and PANC-1 were used in this study. The source and incubation condition of the cells was described previously (27).

### Western Blot Assay

Western blot was performed as previously described (27). Samples of equivalent total protein (40 μg) were loaded. Primary antibody against REST, p-Raf-1, p-beta-catenin, p-MEK-2, p-ERK-1/2 (Abcam, Cambridge, UK, 1:500), E-cadherin, N-cadherin, Vimentin, MMP-9, ZO-1, MEK-2, ERK1/2, Raf-1, beta-catenin, beta-Actin, and Histone H3 (ProteinTech Group, Rosemont, USA, 1:500) were used. Suppression of MAPK signaling pathway with U0126 was performed as previously described (28). In brief, cells incubated in t25 culture flask with 60~70% confluency were treated with 10 μM U0126 for 36 h before further analysis.

### Immunohistochemistry Assay

Immunohistochemical staining of paraffin-embedded pancreatic cancer tissue sections was performed as previously described (27). In brief, immunohistochemical staining of tumor tissue was performed according to the kit manufacturer's protocol (Zhongshan Golden Bridge, Beijing, China) on 4% paraformaldehyde-fixed, paraffin-embedded 4 μm tissue sections. The sections were dried at 60°C for 2 h before de-waxed in xylene and rehydrated by graded alcohols. The sections were incubated in 3% hydrogen peroxide for 12 min to block the endogenous peroxidase activity. The antigens were restored in Tris-EDTA (pH 9.0)/0.01 M sodium citrate buffer (pH 6.0) heated by a microwave oven for 3 min in high heat then 7 min in low heat. Non-specific staining was prevented by blocking sections with 10% goat serum for 15 min. Sections were incubated with REST primary antibodies at the dilution concentration of 1:300 overnight at 4°C in a black humidified box. Then the sections were washed with PBS and incubated with secondary antibodies. Antibody binding was detected by DAB solution. Detection reaction was stopped by washing in distilled water once the sections showed brown color. Finally, sections were counter-stained with hematoxylin for 10 min then evaluated by two different specialists. The expression was scored with the following standard: intensity scores (0-negative, 1-weak, 2-moderate, 3-strong) × positive reaction scores (0~5%-1, 5~25%-2, 25~75%-3, >75%-4). Final score 0–7 was considered as low expression and 8–12 was considered as high expression. Survival analysis (log-rank) of groups with different expression level of REST was performed and visualized with GraphPad Prism 7.

### Stable Transfection With shRNA Plasmid and Lentiviral Vector

shRNA plasmid specific for REST, scrambled shRNA, and lentiviral vector were purchased from GeneChem (GeneChem, Shanghai, China). Stable transfection with lentiviral vector was performed in accordance with manufacturer's protocols. Scrambled shRNA was used as control.

### *In vitro* Proliferation, Migration, and Invasion Assay

Cell proliferation assay, wound healing migration assay and Transwell invasion assay were performed as previously described (27).

### Immunofluorescence Assay

Cells cultured on cover slides were fixed with 4% paraformaldehyde at room temperature for 30 min before they were incubated in blocking buffer (4% BSA in PBS) at room temperature for another 30 min. Blocked cells were incubated with E-cadherin and N-cadherin antibodies (ProteinTech Group, 1:100) at 4°C overnight before they were incubated with Fluorescein (FITC) conjugated anti-mouse IgG and Rhodamine (TRITC) conjugated anti-mouse IgG (ProteinTech Group, 1:100). The nucleus was stained with DAPI and the cells were visualized with confocal microscope (Nikon, Tokyo, Japan).

### Animal Studies

Subcutaneous tumor models (five nude mouse per group) were established with 4-week-old female BALB/c nude mice (Huafukang Biotechnology, Beijing, China) as previously described (27). Metastatic tumor model (three nude mouse per group) of pancreatic cancer was established by superior mesenteric vein injection on 4 weeks old female BALB/c nude mice (Huafukang Biotechnology). A total number of 5 × 10^6^ AsPC-1/REST knockdown AsPC-1 cells suspended in 0.15 ml PBS was injected. Nude mice were kept in specific pathogen free level animal laboratory of the Shengjing Hospital for another 21 days (subcutaneous tumor model) and 14 days (metastasis tumor model) before sacrifice.

### Statistical Analysis

Student's *t*-test was calculated with the IBM SPSS Statistics version 19.0. Log-rank test was calculated with Survival curve function of the GraphPad Prism 7. *p* < 0.05 was considered statistically significant in this study.

## Results

### Identification of REST as the Hub Gene for Cancer Stage With WGCNA Analysis

**Figure 1:**
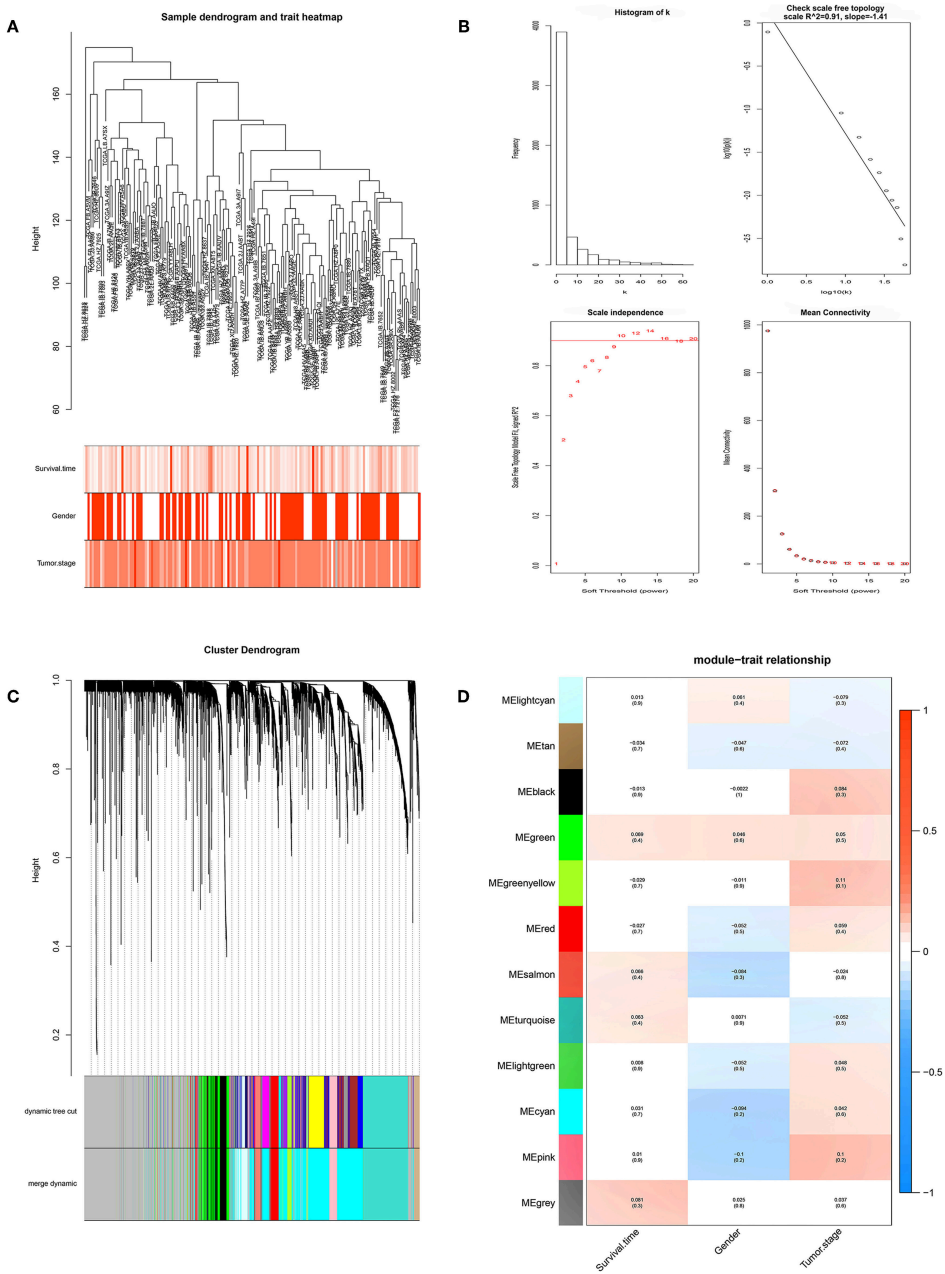
Identification of clinically significant modules for survival time and tumor stage of pancreatic cancer. **(A)** Clustering dendrogram of pancreatic cancer samples in the TCGA database. **(B)** Determination of soft-thresholding power (*p* = 10). **(C)** Gene hierarchical clustering dendrogram of identified gene modules. **(D)** Heat map of identified functional gene modules and corresponding clinical traits. Number without bracktes indicates the correlation score and number in the brackets indicates the *p* value.

**Figure 2:**
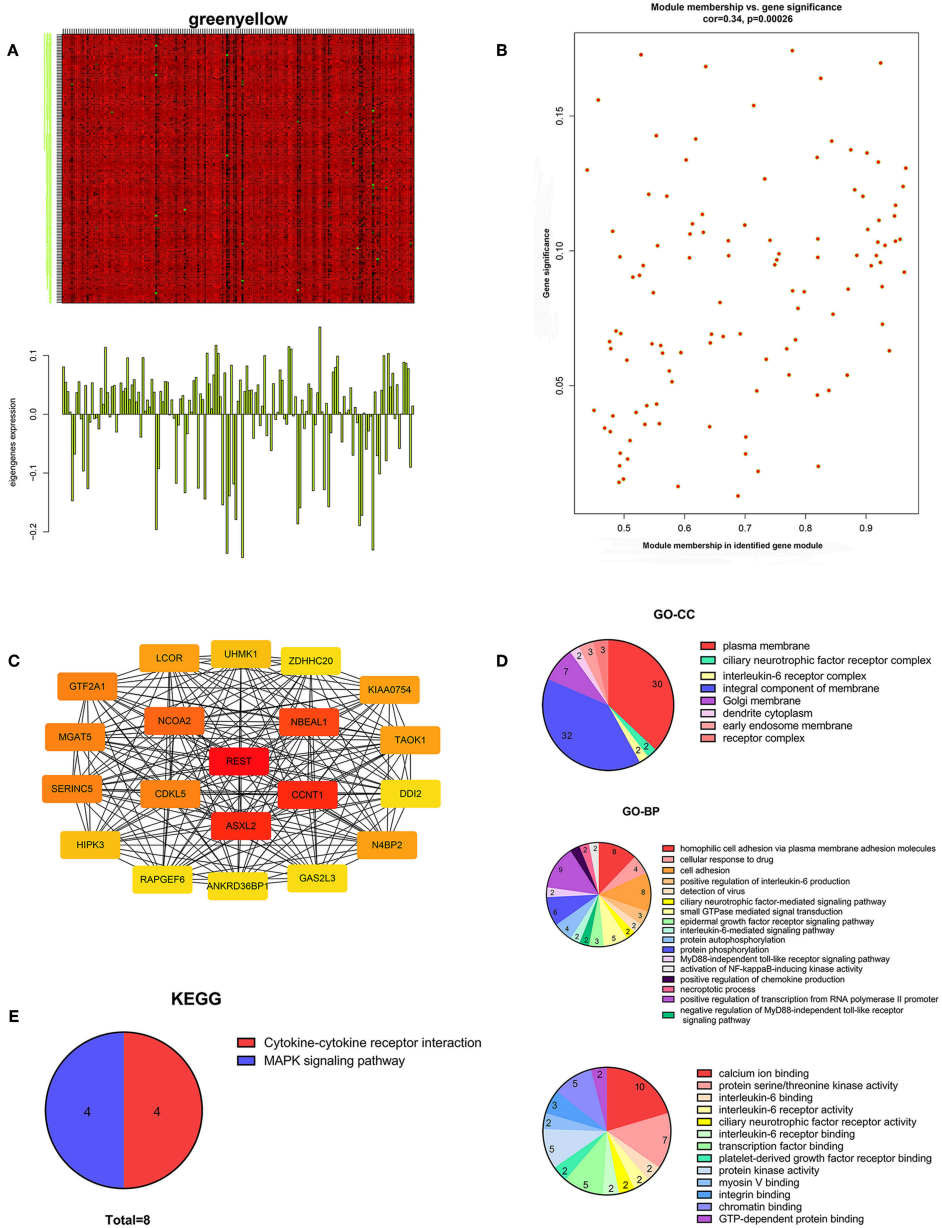
Characterization of the key gene module for pancreatic cancer stage. **(A)** Heatmap of individual gene expression level in greenyellow module. **(B)** Individual gene significance with tumor stage in greenyellow module. **(C)** Protein-protein interaction network of the top 20 inter-connected genes in greenyellow module. **(D)** Gene Ontology analysis of greenyellow module. **(E)** Kyoto Encyclopedia of Genes and Genomes pathway analysis of greenyellow module.

We performed WGCNA analysis with data from the TCGA database to identify the key gene module which is closely associated with the prognosis of pancreatic cancer (Figures 1A,B). Eleven functional gene modules were identified in which the greenyellow module showed strongest correlation with tumor stage and was chosen for further analysis (Figures 1C,D). Expression level and individual gene significance of the greenyellow module was visualized (Figures 2A,B). PPI network analysis of the top 20 genes with the highest intramodular connectivity in greenyellow module identified REST as the hub gene (Figure 2C). GO analysis and KEGG pathway analysis of the greenyellow module showed that the module was enriched in genes regulating cancer metastasis and MAPK signaling pathway (Figures 2D,E).

### Expression Level Validation and Survival Analysis of Rest in Pancreatic Cancer

**Figure 3:**
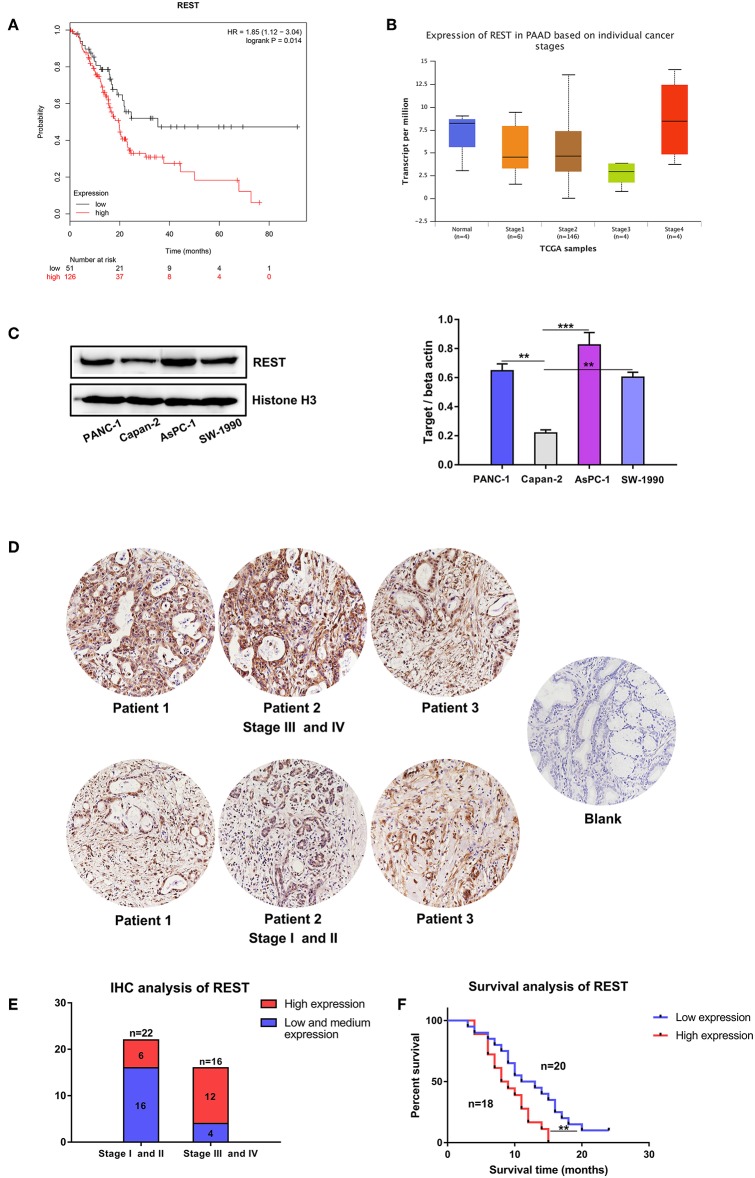
Expression level validation and survival analysis of REST in pancreatic cancer. **(A)** Survival analysis of patients with different expression level of REST in the TCGA database. **(B)** Expression level of REST in different individual pancreatic cancer stages in the TCGA database. **(C)** Expression level of REST in human pancreatic cancer cell lines with different metastatic phenotype. **(D)** Representative images of immunohistochemical staining for REST in pancreatic cancer tissue sections with different stages (magnification, ×200). **(E)** Expression level of REST in 38 pancreatic cancer tissue sections with different stages. **(F)** Survival analysis of 38 pancreatic cancer patients with different expression level of REST. *p* < 0.05 marked ^*^p < 0.01 marked ^**^p < 0.001 marked^***^.

Survival analysis of REST with clinical data from the TCGA database suggested worse overall survival rate of patients with higher expression level of REST (Figure 3A). Expression level validation of REST in different cancer stages with the TCGA showed higher expression level of REST in advanced stage than early stage (Figure 3B). Validation with human pancreatic cancer cell lines (AsPC-1, PACN-1, SW-1990 for metastatic phenotype and Capan-2 for non-metastatic phenotype) and pancreatic cancer tissue sections showed that the expression level of REST was higher in advanced pancreatic cancer tissue samples and metastatic phenotype cell lines (Figures 3C–E, Table 1). Furthermore, patients with higher expression level of REST showed worse overall survival rate (Figure 3F).

**Table 1:** Clinical characteristics of 38 qualified pancreatic cancer tissue sections.

**Characteristics**	**Number**	**Percentage (%)**
**Gender**		
Male	21	55
Female	17	45
**Age**		
Mean (years)	58.36 ± 9.48	
**Histopathologic subtype**		
Well-differentiated adenocarcinomas	17	45
Moderately differentiated adenocarcinomas	8	21
Poorly differentiated adenocarcinomas	13	34
**TNM staging**		
T2N0M0	9	24
T2N1M0	3	8
T2N1M1	1	3
T3N0M0	11	28
T3N1M0	6	16
T3N0M1	4	10
T3N1M1	2	5
T4N0M0	1	3
T4N1M0	1	3
**Local invasion and distant metastasis**		
Local invasion	11	28
Regional lymph node metastasis	13	34
Liver metastasis	5	13
Ascites metastasis	2	5

### REST Knockdown Suppress Metastatic Phenotype of Pancreatic Cancer Cells *in vitro*

**Figure 4:**
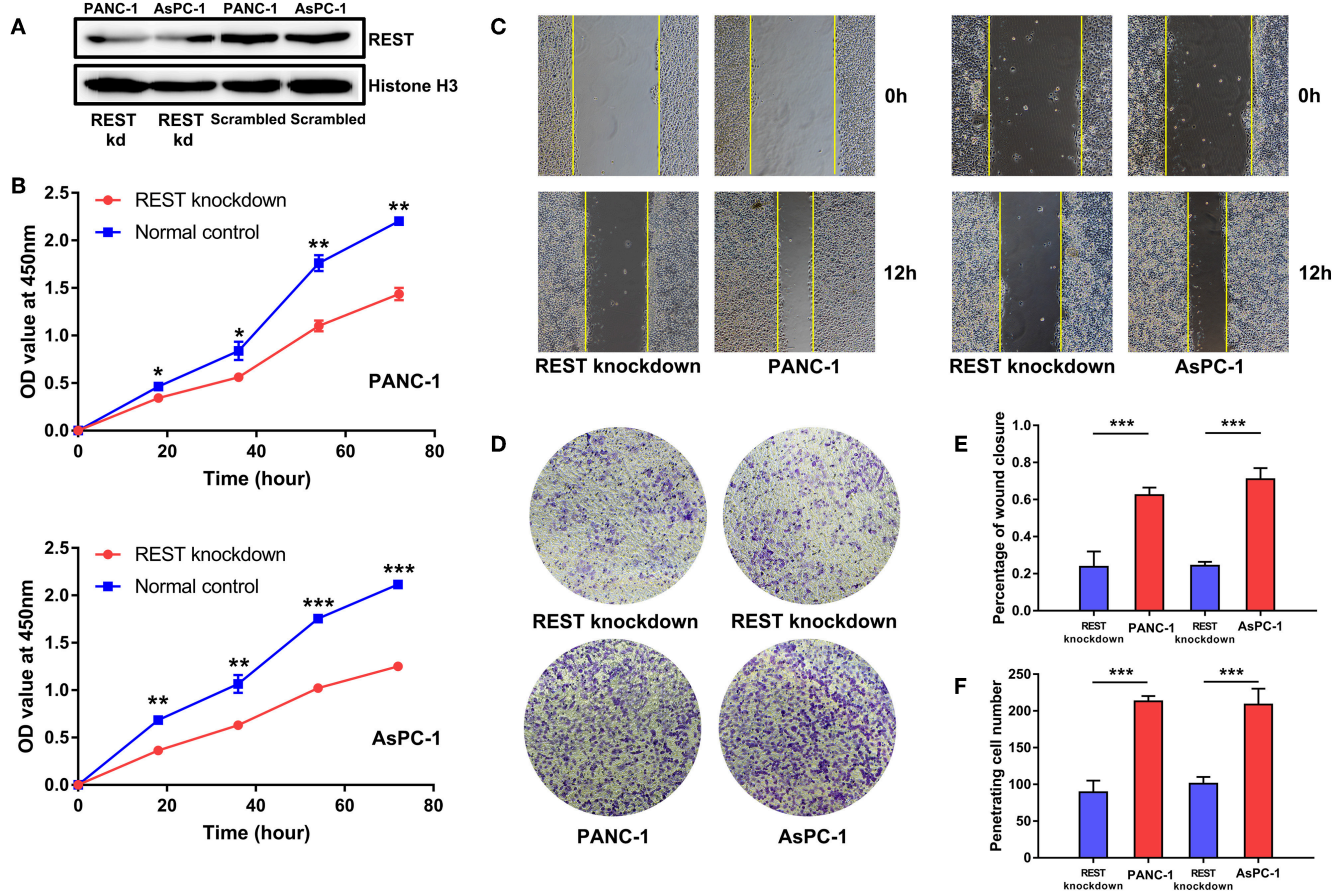
Knockdown of REST suppress proliferation, migration, and invasion capabilities of pancreatic cancer cell lines *in vitro*. **(A)** Knockdown of REST with stable transfection in PANC-1 and AsPC-1 cell lines. **(B)** Knockdown of REST suppress proliferation capability in PANC-1 and AsPC-1 cell lines. **(C)** Knockdown of REST suppress migration capability in PANC-1 and AsPC-1 cell lines (magnification, × 100). **(D)** Knockdown of REST suppress invasion capability in PANC-1 and AsPC-1 cell lines (magnification, × 100). **(E)** Statistical analysis of migration assay. **(F)** Statistical analysis of invasion assay. *p* < 0.05 marked ^*^p < 0.01 marked ^**^p < 0.001 marked^***^.

**Figure 5:**
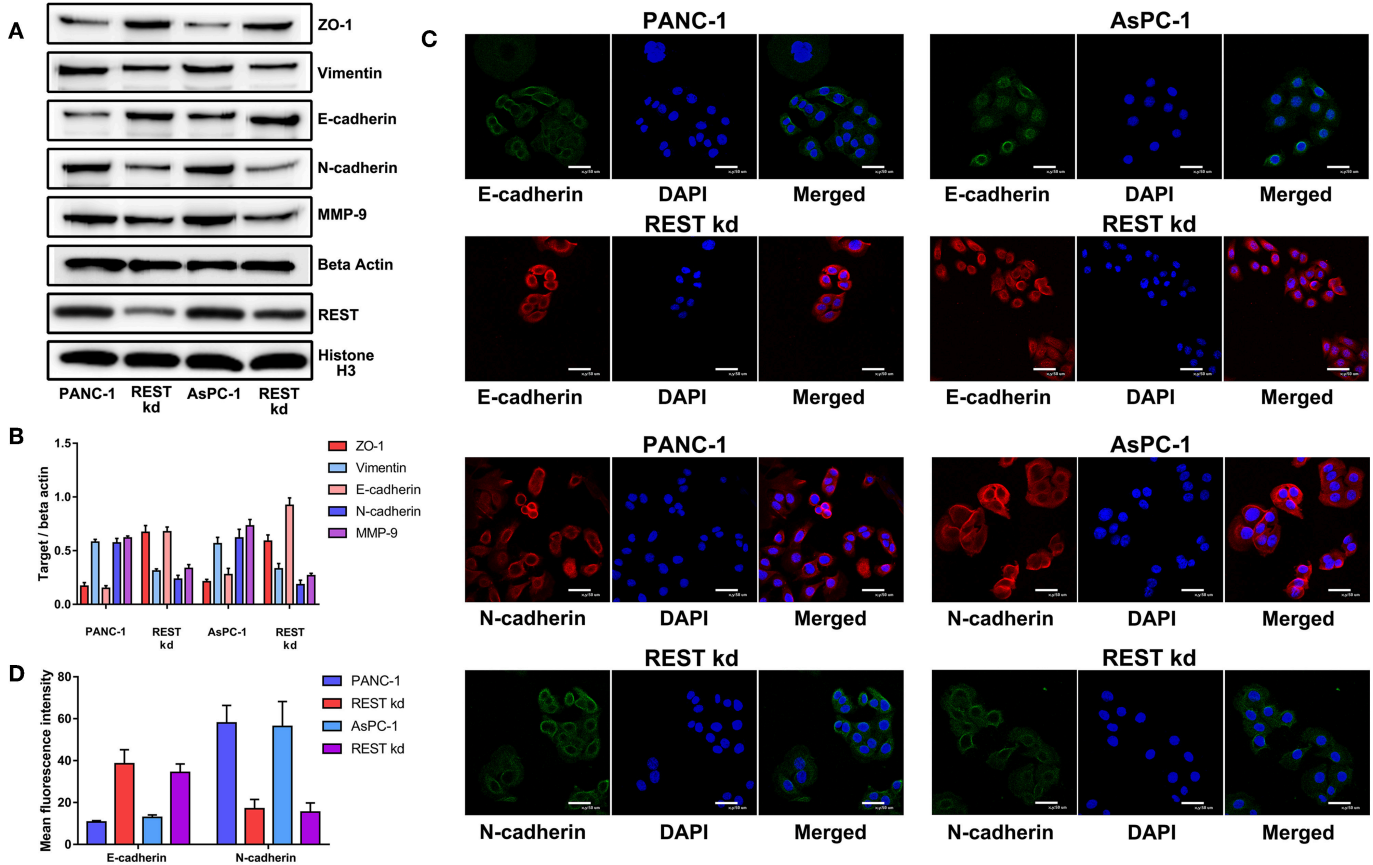
Knockdown of REST suppress epithelial-mesenchymal transition of pancreatic cancer cells *in vitro*. **(A)** Representative Western blot images of changes in the expression level of mesenchymal (N-cadherin, Vimentin and MMP-9) and epithelial (E-cadherin and ZO-1) markers after REST knockdown. **(B)** Bar chart of changes in the expression level of mesenchymal and epithelial markers **(C)** Representative confocal microscope images of changes in the expression level of mesenchymal (N-cadherin) and epithelial (E-cadherin) markers after REST knockdown (magnification, ×200). **(D)** Bar chart of changes in the expression level of mesenchymal and epithelial markers.

To investigate the potential role of REST in the metastasis of pancreatic cancer, we knocked down the expression of REST in PANC-1 and AsPC-1 cell lines (Figure 4A). *In vitro* proliferation, migration and invasion assay showed that knockdown of REST suppressed the proliferation, migration and invasion capabilities of PANC-1 and AsPC-1 cells (Figures 4B–F). These results suggested that knockdown of REST could suppress the metastatic phenotype of these two pancreatic cancer cell lines. Next, we investigated the role of REST in EMT as it is the primal step for cancer cells' invasion and metastasis. Western blot analysis of the EMT markers showed that knockdown of REST decreased the expression level of mesenchymal markers like N-cadherin, Vimentin and MMP-9 while increased the expression level of epithelial markers like E-cadherin and ZO-1 (Figures 5A,B). Further immunofluorescence assay showed consistent results as knockdown of REST decreased expression level of N-cadherin and increased expression level of E-cadherin (Figures 5C,D). These results suggested that REST may promote EMT in pancreatic cancer cells.

### REST Knockdown Suppress Xenograft Tumor Growth and Metastasis *in vivo*

**Figure 6:**
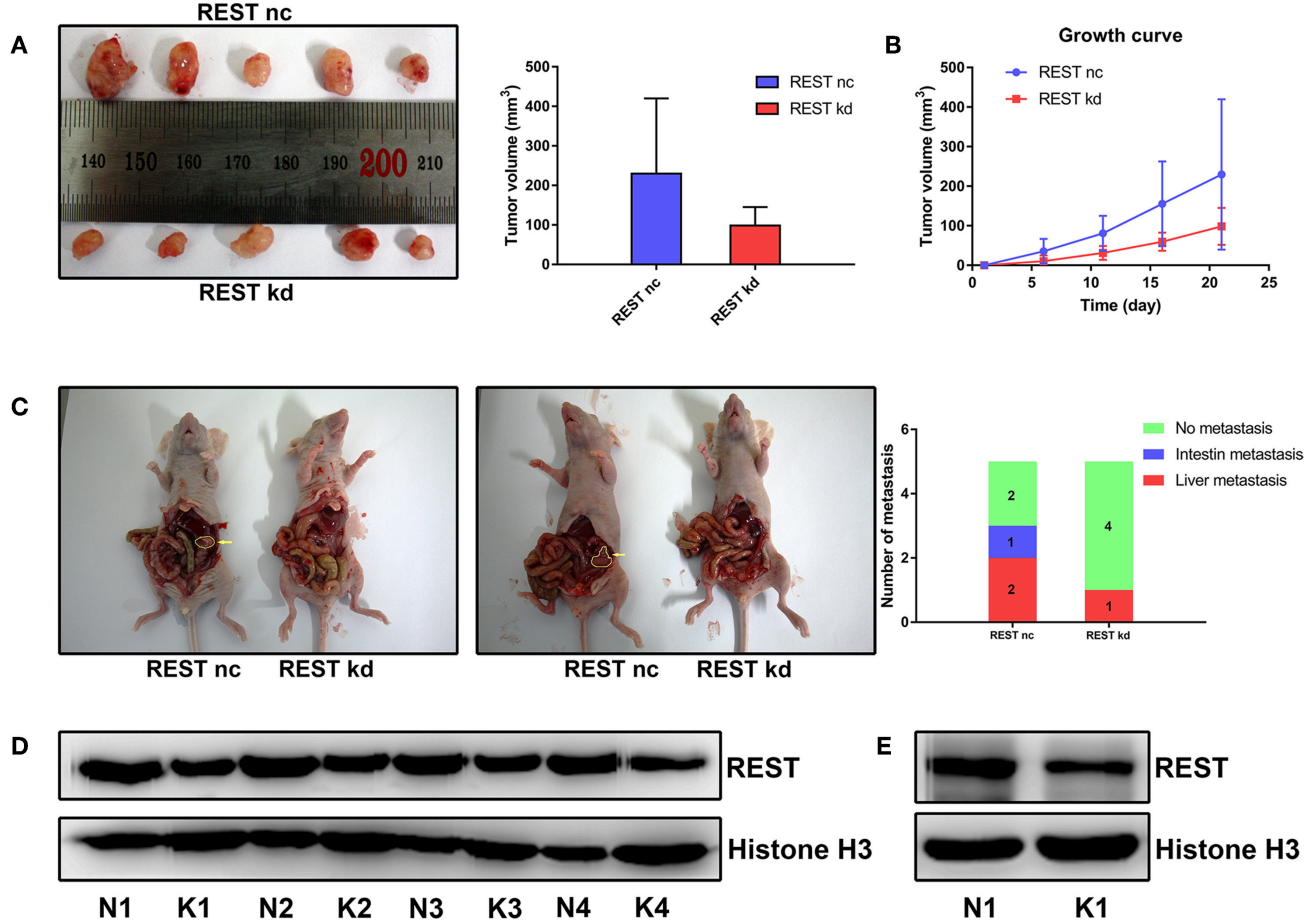
Knockdown of REST suppress xenograft tumor growth and metastasis *in vivo*. **(A)** Images of resected subcutaneous tumor after 21 days of implantation. **(B)** Growth curve of implanted subcutaneous tumor. **(C)** Representative images of liver metastasis and intestine metastasis of superior mesenteric vein injection. The arrow indicates the metastatic tumor. **(D)** Western blot analysis of REST in resected subcutaneous tumor. N for normal control and K for REST knockdown. **(E)** Western blot analysis of REST in rescted metastatic tumor. N for normal control and K for REST knockdown.

To further validate the results of *in vitro* experiments, we established subcutaneous tumor model and metastatic tumor model of pancreatic cancer in nude mice. Results of subcutaneous tumor model showed that knockdown of REST suppressed xenograft tumor growth *in vivo* (Figures 6A,B,D). Results of metastatic tumor model showed that knockdown of REST suppressed the liver metastasis and intestine metastasis (Figures 6C,E). These *in vitro* and *in vivo* results suggested that REST may function as a key promoter for pancreatic cancer metastasis.

### REST May Serve as a Novel Cross Point of MAPK and Wnt/beta-catenin Signaling Pathway in Pancreatic Cancer

**Figure 7:**
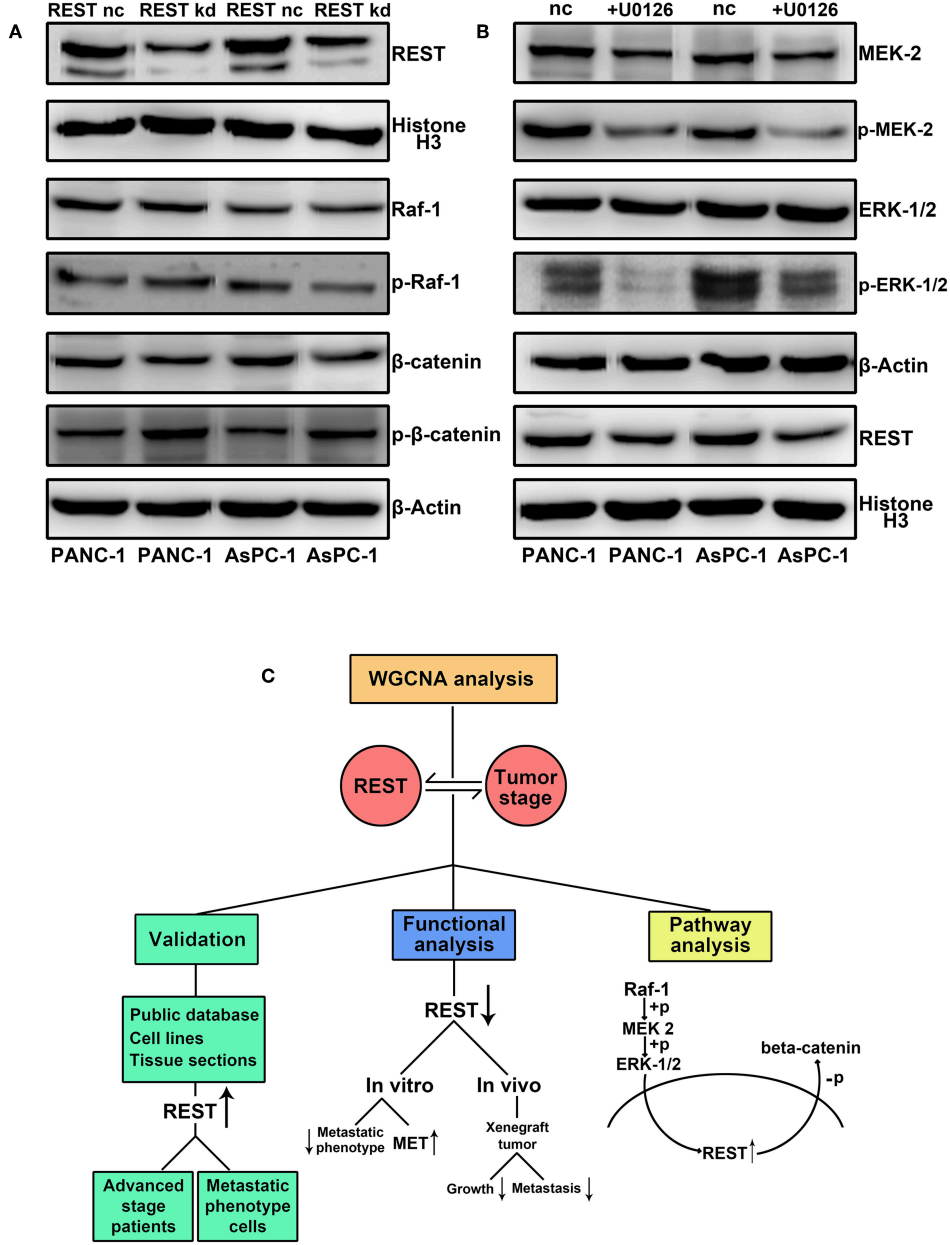
REST may serve as a novel cross point of MAPK and Wnt/beta-catenin signaling pathway in pancreatic cancer. **(A)** REST knockdown increase the phosphorylation level of beta-catenin while not affect the expression and phosphorylation level of Raf-1. **(B)** Suppression of MAPK signaling pathway with U0126 decrease the expression level of REST. **(C)** Flowchart of the current study.

Finally, we investigated the potential correlation of REST with MAPK and Wnt/beta-catenin signaling pathway in pancreatic cancer. Knockdown of REST increased the phosphorylation level of beta-catenin (Figure 7A). However, knockdown of REST didn't affect the expression or phosphorylation level of Raf-1 (Figure 7A). Suppression of MAPK signaling pathway with U0126 decreased the expression level of REST (Figure 7B). Based on these results, we speculated that REST might be a novel cross point of MAPK and Wnt/beta-catenin signaling pathway (Figure 7C).

## Discussion

Multiple studies and databases like the TCGA database have provided and collected numerous genomic and clinical data aiming to further elucidate the progression of cancer (29). Previous studies focused on data mining of the aforementioned genomic data to identify the differentially expressed genes (DEGs) in cancer progression (30). Indeed, these identified DEGs hold promising potential to serve as key regulators in cancer progression and novel predictive biomarkers for cancer detection and prognosis (31). However, these studies didn't fully utilized the aforementioned clinical data as they didn't correlate the identified DEGs with certain clinical traits. Therefore, in this study, we utilized WGCNA which is able to identify functional gene modules and further correlate them with corresponding clinical phenotype. We identified a key gene module which has the strongest association with cancer stage of pancreatic cancer. Further analysis identified REST as the hub gene of this gene module. These results led to the assumption that REST might be one of the key promoters for pancreatic cancer's metastasis as patients of advanced stage are usually characterized by extensive local invasion and distant metastasis. Validation with public database and our own patient samples showed higher expression level of REST in patients with advanced cancer stage. Further *in vitro* and *in vivo* functional experiments showed that knockdown of REST suppressed growth and metastasis of pancreatic cancer cell lines and xenograft tumors. Combing results of the present study and aforementioned studies, we speculated the existence of a pancreatic cancer specific splice variant of REST. This specific isoform of REST holds promising potential to serve as a specific prognostic biomarker and a sensitive therapy target for pancreatic cancer. This speculation requires further validation with experiments in our future studies. What's more, we only analyzed the indirect relationship between REST and MAPK signaling pathway in this study. Further investigation on the direct molecular mechanism of REST and MAPK signaling pathway is required in our future studies.

## Ethics Statement

The current study was approved by local ethics committee of the affiliated Shengjing Hospital of China Medical University (animal studies-2017PS006K, tissue sample collection-2017PS24K). All animal handling procedures were performed in accordance with standard procedures of the local ethics committee. Thirty-eight qualified paraffin-embedded pancreatic cancer tissue sections with follow-up data were collected from the local pathological department of Shengjing Hospital between 2014 and 2016.

## Author Contributions

HJ performed most of the experiments and wrote the paper. PL, LK, XF, YG, TW, and DS performed some of the experiments and analyzed the data. XT designed the study and revise the paper.

### Conflict of Interest Statement

The authors declare that the research was conducted in the absence of any commercial or financial relationships that could be construed as a potential conflict of interest.
